# Spontaneous trigger words associated with confirmed out-of-hospital cardiac arrest: a descriptive pilot study of emergency calls

**DOI:** 10.1186/s13049-019-0696-1

**Published:** 2020-01-03

**Authors:** Joonas Tamminen, Erik Lydén, Jan Kurki, Heini Huhtala, Antti Kämäräinen, Sanna Hoppu

**Affiliations:** 1 Faculty of Medicine and Health Technology, Tampere University, PO Box 2000, FI-33520 Tampere, Finland; 2 Emergency Medical Services, Tampere University Hospital, PO Box 2000, FI-33521 Tampere, Finland; 3 Biostatistics, Faculty of Social Sciences, Tampere University, FI-33014 Tampere, Finland

**Keywords:** Out-of-hospital cardiac arrest, Bystander cardiopulmonary resuscitation, Dispatch, Emergency calls, Trigger words

## Abstract

**Background:**

According to the International Liaison Committee on Resuscitation (ILCOR), the trigger words used by callers that are associated with cardiac arrest constitute a scientific knowledge gap. This study was designed to find hypothetical trigger words in emergency calls in order to improve the specificity of out-of-hospital cardiac arrest recognition.

**Methods:**

In this descriptive pilot study conducted in a Finnish hospital district, linguistic contents of 80 emergency calls of dispatcher-suspected or EMS-encountered out-of-hospital cardiac arrests between January 1, 2017 and May 31, 2017 were analysed. Spontaneous trigger words used by callers were transcribed and grouped into 36 categories. The association between the spontaneous trigger words and confirmed true cardiac arrests was tested with logistic regression.

**Results:**

Of the suspected cardiac arrests, 51 (64%) were confirmed as true cardiac arrests when ambulance personnel met the patient. A total of 291 spontaneous trigger words were analysed. ‘Is not breathing’ (*n* = 9 [18%] in the true cardiac arrest group vs *n* = 1 [3%] in the non-cardiac arrest group, odds ratio [OR] 6.00, 95% confidence interval [CI] 0.72–50.0), ‘the patient is blue’ (*n* = 9 [18%] vs *n* = 1 [3%], OR 6.00, 95% CI 0.72–50.0), ‘collapsed or fallen down’ (*n* = 12 [24%] vs *n* = 2 [7%], OR 4.15, 95% CI 0.86–20.1) and ‘is wheezing’ (*n* = 17 [33%] vs *n* = 5 [17%], OR 2.40, 95% CI 0.78–7.40) were frequently used to describe true cardiac arrest. ‘Is snoring’ was associated with a false suspicion of cardiac arrest (*n* = 1 [2%] vs *n* = 6 [21%], OR 0.08, 95% CI 0.009–0.67).

**Conclusions:**

In our pilot study, no trigger word was associated with confirmed cardiac arrest. ‘Is wheezing’ was a frequently used spontaneous trigger word among later confirmed cardiac arrest victims.

## Background

Survival after out-of-hospital cardiac arrest (OHCA) remains modest despite standardised dispatch protocols in emergency medical services (EMS) systems, increased community training and the introduction of post-resuscitation care [1–3]. Nevertheless, early pre-hospital interventions do have a substantial impact on the survival of OHCA victims. Bystander-initiated cardiopulmonary resuscitation (CPR) increases the chances of 30-day survival twofold and is associated with improved long-term neurological outcome [4, 5].

Early recognition of cardiac arrest is the cornerstone of the chain of survival [6–8]. The well-known clinical signs of cardiac arrest are unresponsiveness and absent or abnormal breathing [6]. However, it is unclear how these signs and symptoms, especially agonal breaths, are interpreted and described by laypeople. Besides cardiac arrest, these clinical signs and symptoms are also related to many other medical conditions, which results in significant amount of false positive suspicions of OHCA. Emergency calls could contain hypothetical trigger words that current dispatch protocol may not recognise; the International Liaison Committee on Resuscitation (ILCOR) has announced that trigger words form a scientific knowledge gap [9]. These trigger words could be used to facilitate recognition of OHCA, to reduce time to dispatch EMS and to increase immediate bystander CPR rates. Importantly, they could be used to reduce the number of false positive alarms and thus to improve the specificity of recognition of cardiac arrest.

To test whether hypothetical trigger words exist and to generate more specific hypotheses, our study was designed as a descriptive pilot study. This pilot study aims to examine the association between true OHCA confirmed by ambulance personnel and laypeople’s spontaneous trigger words regarding physiological deterioration of a patient in the context of emergency-dispatcher-suspected or EMS-encountered OHCA.

## Methods

This descriptive pilot study was conducted in the Pirkanmaa Hospital District, Finland, which serves the city of Tampere and a surrounding rural area covering a population of 510,000 [10]. In the study area, emergency calls are processed by trained emergency dispatchers, majority of whom are not medical professionals. The length of the formal dispatcher education is 1.5 years in Finland [11]. The national call processing is protocol-based and computer-aided. Recognition of cardiac arrest is based on three questions: (1) Tell me exactly what happened, (2) Is she/he conscious? and (3) Is she/he breathing normally? [11] During the study period, the emergency dispatcher did not receive any additional feedback that differed from the standard quality control.

Between January 1, 2017 and May 31, 2017, all audio recordings and electronic mission reports of consecutive emergency calls of dispatcher-suspected OHCA or EMS-encountered OHCA that a dispatcher had not suspected in the study area were extracted from the EinsatzLeitSystem (ELS) database maintained by the Emergency Response Centre Agency [12]. As the aim of the study was to address laypeople’s interpretations of physiological deterioration of an OHCA patient, cases with unwitnessed OHCA, traumatic cause for OHCA or an institutional resuscitation attempt were excluded.

As the study was retrospective and based on registry data only, with no interventions or patient contact involved, the need for patient consent was waived. The study protocol was approved by the institutional review board of the Pirkanmaa Health District (R17156, November 7th, 2017).

### Spontaneous trigger words

Spontaneous speech, defined as something that the caller said without being prompted or asked by the dispatcher, was transcribed by authors EL and JK who are professional paramedics. Caller’s whole answer to a preceding question was considered as non-spontaneous speech regardless of the duration or the length of the answer. In order to analyse transcribed speech, different words with the same semantic meaning were put in a single category [13, 14]. Authors JT, EL and JK interpreted the semantic meaning of trigger words and categorised them. The basis of our categorisation was a word list introduced by Berdowski et al. [7], which included seven categories: breathing, consciousness, facial colour, death, heart problems, resuscitation, and other. In addition, the ABCDE approach was used to formulate our categorisation [15]. The ABCDE is a mnemonic for a generally accepted treatment protocol for critically ill patients. In our study, the spontaneous trigger words were grouped into seven main categories and thirty-six subcategories, the former of which included altered level of unconsciousness, death, breathing, circulation, disability, history of present illness, and unclassified. Our circulation category included facial colour and heart problems as subcategories. In cases of an ambiguous trigger word, the other two authors verified the suggested trigger word category.

Each emergency call could fulfil the criteria of each subcategory once. Subsequently, two or more trigger words were counted as a duplicate if the caller repeated the same word or if the caller used words that had a different linguistic form but had an identical semantic meaning. Ultimately, the trigger words were translated from Finnish to English (United Kingdom) by two native Finland linguists who have MA degrees in communication sciences.

### Confirmation of true cardiac arrest

The trigger words were stratified into true cardiac arrest and non-cardiac arrest groups. The mission reports were used to identify true cardiac arrests, as there was no national cardiac arrest registry in Finland. After each mission, the EMS personnel filled out specific documentation that contained dispatch and transportation codes (e.g. the patient is confirmed dead, or the patient had return of spontaneous circulation, or CPR was being performed during transportation or the patient had had any other medical emergency). The true cardiac arrest events were confirmed by the EMS personnel based on these documentations. A transportation code for a non-OHCA event could be, for example, rhythm disturbance or intoxication.

### Statistical methods

SPSS software version 25 (SPSS Inc., Chicago, IL, USA) was used to perform the statistical calculations. Categorical and continuous variables were reported as frequencies and proportions and as medians and interquartile ranges, respectively. The comparison between the groups was performed using a χ^2^ or a two-tailed Fisher’s exact test for the categorical data and a Mann–Whitney U-test for the continuous, nonparametric data. A univariate logistic regression was used to assess the association between the spontaneous trigger words and confirmed cardiac arrests, and the results were presented as odds ratio (OR) with 95% confidence interval (CI). A two-sided *p*-value < 0.05 was considered statistically significant.

## Results

**Fig. 1:**
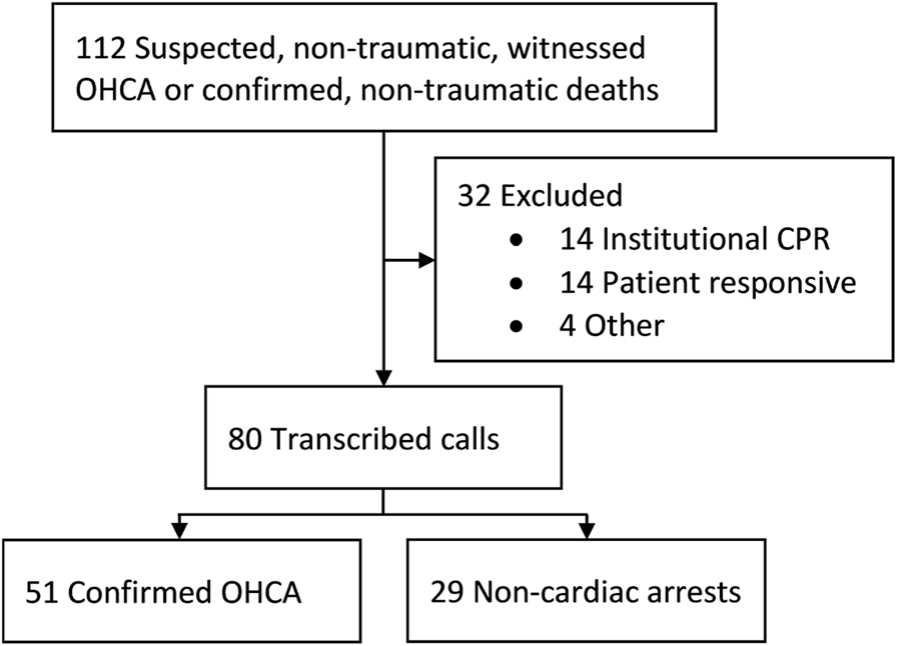
Study population flowchart

During the study period, 112 emergency calls met our inclusion criteria. A total of 32 (29%) cases were excluded because they related to an institutional resuscitation, the patient was awake or other reasons (e.g. poor sound quality), and 80 (71%) emergency calls were transcribed as presented in Fig. 1. Of the suspected cardiac arrests, 51 (64%) were confirmed as true cardiac arrests, and 29 (36%) of the suspected cardiac arrests were regarded as non-cardiac arrest events when EMS evaluated the patient.

**Fig. 2:**
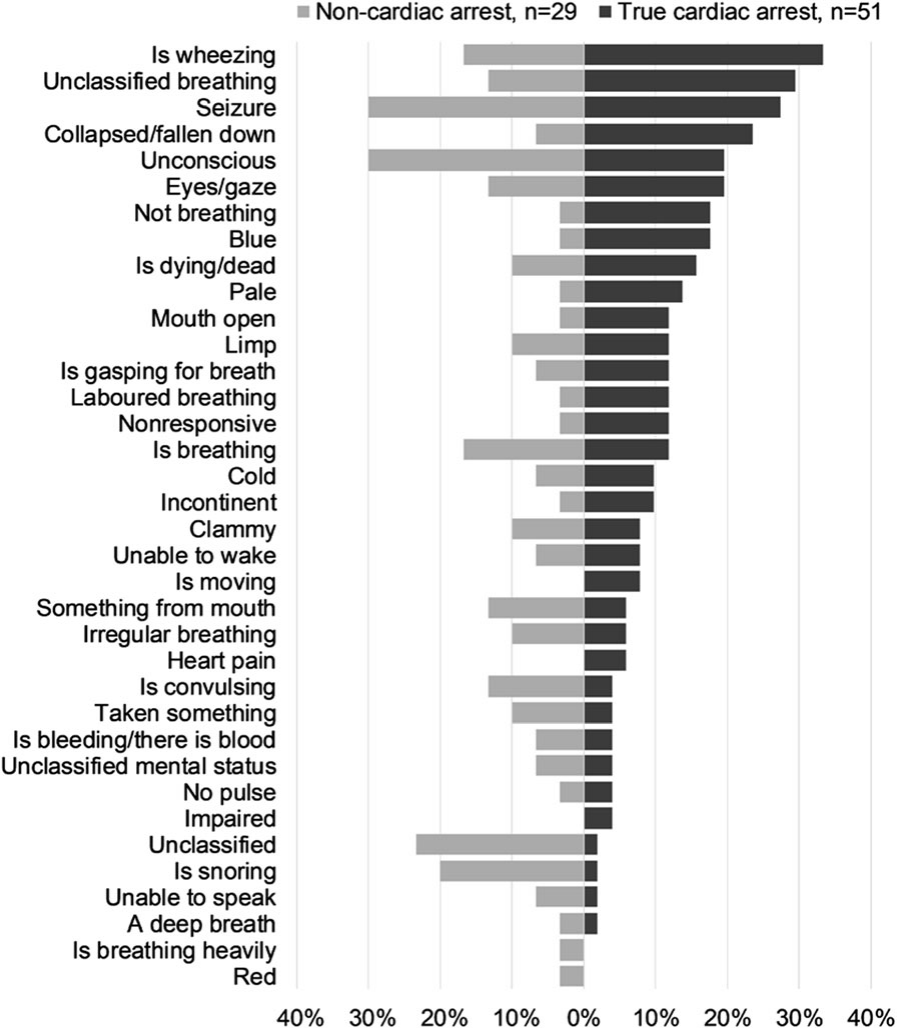
The observed 291 spontaneous trigger words in 36 categories

The emergency call and mission characteristics are presented in Table 1. Most cardiac arrests were suspected after an ambulance was dispatched, and two confirmed cardiac arrests were not recognised by the dispatcher. The time of OHCA suspicion, the number of trigger words and the duration of speech intervals were similar between the groups. A total of 291 spontaneous trigger words were analysed; 93 (32%) and 41 (14%) of them concerned breathing and altered level of consciousness, respectively. The distribution of spontaneous trigger words in confirmed cardiac arrest and non-cardiac groups is presented in Fig. 2.

**Table 1:** Emergency call and mission characteristics

	True cardiac arrest	Non-cardiac arrest	*p*-value
	*n* = 51	*n* = 29	
Trigger words			
Total, n (%)	194 (67)	97 (33)	
Median (IQR)	3 (2–5)	3 (2–5)	0.369
Time of OHCA suspicion, n = (%)			
Initial reason for dispatch	15 (29)	6 (21)	0.440
EMS en route	34 (67)	23 (79)	0.307
OHCA not suspected	2 (4)	0 (0)	0.532
Initial dispatch code non-specific	23 (45)	12 (41)	0.817
Duration, median (IQR); min:sec			
Emergency call	6:47 (5:12–8:35)	5:31 (3:41–8:49)	0.423
Total spontaneous speech	5:12 (3:31–6:44)	3:55 (3:05–7:08)	0.506
Initial description of situation	0:05 (0:04–0:10)	0:05 (0:04–0:09)	0.590

*IQR* interquartile range; *OHCA* out-of-hospital cardiac arrest; *EMS* emergency medical services

The results of the univariate logistic regression are shown in Table 2. The spontaneous trigger words that were more frequently used to describe true cardiac arrest were ‘is not breathing’ (*n* = 9 [18%] in the true cardiac arrest group vs *n* = 1 [3%] in the non-cardiac arrest group, odds ratio [OR] 6.00, 95% confidence interval [CI] 0.72–50.0), ‘the patient is blue’ (n = 9 [18%] vs *n* = 1 [3%], OR 6.00, 95% CI 0.72–50.0), ‘collapsed or fallen down’ (*n* = 12 [24%] vs *n* = 2 [7%], OR 4.15, 95% CI 0.31–20.1) and ‘is wheezing’ (*n* = 17 [33%] vs *n* = 5 [17%], OR 2.40, 95% CI 0.78–7.40). ‘Is snoring’ was associated with a false suspicion of cardiac arrest (n = 1 [2%] vs *n* = 6 [21%], OR 0.08, 95% CI 0.009–0.67).

**Table 2:** Distribution (%) of the spontaneous trigger words and their association with confirmed cardiac arrests

	True cardiac arrest	Non-cardiac arrest	OR (95% CI)
Trigger words	*n* = 51	*n* = 29	
Dead or is dying	16	10	1.61 (0.39–6.63)
Altered level of consciousness			
Unconscious	20	31	0.54 (0.19–1.55)
Unable to wake	8	7	1.15 (0.20–6.69)
Nonresponsive	12	3	3.73 (0.43–32.7)
Impaired	4	0	NA
Unable to speak	2	7	0.27 (0.02–3.12)
Unclassified consciousness	4	7	0.55 (0.07–4.14)
Breathing			
Is breathing	12	17	0.42 (0.12–1.51)
Not breathing	18	3	6.00 (0.72–50.0)
Laboured	12	3	3.73 (0.43–32.7)
Heavily	0	3	NA
Irregularly	6	10	0.54 (0.10–2.88)
Is gasping for breath	12	7	1.80 (0.34–9.56)
A deep breath	2	3	0.56 (0.03–9.30)
Is snoring	2	21	0.08 (0.009–0.67)
Is wheezing	33	17	2.40 (0.78–7.40)
Unclassified breathing	29	14	2.60 (0.77–8.78)
Circulation			
No pulse	4	3	1.14 (0.10–13.2)
Pale	14	3	4.46 (0.52–38.2)
Red	0	1	NA
Blue	18	3	6.00 (0.72–50.0)
Cold	10	7	1.47 (0.27–8.09)
Clammy	8	10	0.74 (0.15–3.55)
Is bleeding or there is blood	4	7	0.55 (0.07–4.14)
Heart pain	6	0	NA
Disability			
Is convulsing	4	14	0.26 (0.04–1.49)
Limp	12	10	1.16 (0.27–5.01)
Incontinent	10	3	3.04 (0.34–27.4)
Eyes or gaze	20	14	1.52 (0.43–5.38)
Mouth open	12	3	3.73 (0.43–32.7)
Something from mouth	6	14	0.39 (0.08–1.88)
Is moving	8	0	NA
History of present illness			
Collapsed or fallen down	24	7	4.15 (0.86–20.1)
Seizure	27	31	0.84 (0.31–2.28)
Taken something	4	10	0.35 (0.06–2.25)
Unclassified	2	24	0.06 (0.007–0.54)

*OR* odds ratio; *CI* confidence interval; *NA* not applicable

## Discussion

In this descriptive pilot study conducted in a Finnish hospital district, the linguistic contents of 80 emergency calls of suspected, non-traumatic, witnessed OHCAs or EMS-encountered, non-traumatic OHCAs that a dispatcher had not suspected were evaluated. The focus of the study was on spontaneous speech used by the caller since it was hypothesised to contain trigger words that the current dispatch protocol may have missed. If recognised, these trigger words could make dispatching faster and more specific. Although ILCOR notes that the trigger words associated with OHCA are a scientific knowledge gap, only one Dutch study has explored trigger words and a couple of Australian studies have examined the communication between emergency dispatchers and laypeople [7, 16, 17].

Our emergency dispatchers performed well during the five-month study period; the emergency dispatcher did not recognise two later confirmed cardiac arrests. The sensitivity for OHCA recognition was 96.2% in our material whereas a recently published systematic review concluded that the global sensitivity for OHCA recognition is 73.9% (range 14.1–96.9%) [18]. The review included three studies conducted in Finnish regions which found slightly lower sensitivities as compared with our results: 82.9, 82.3 and 79.4%, respectively [11, 19, 20]. As Viereck et al. argue, the definition of a recognised cardiac arrest is ambiguous and may result in conflicting estimates of the performance of a given EMS system.

According to the European Resuscitation Council (ERC) guidelines, recognition of OHCA is based on the combination of the patient being recognised as unconscious and apnoeic or breathing abnormally. One might argue that interpretation of the trigger words in relation to breathing is conditional on what is said about the conscious state and vice versa. However, we postulate that an individual trigger may combine the semantic information regarding both level of consciousness and breathing in the context of a medical emergency.

In our material, there were two important trigger words in the breathing category worth noting: ‘is wheezing’ (Finnish: *korisee*) and ‘is snoring’ (Finnish: *kuorsaa*). The former does not mean obstructive wheezing but rather a death rattle or choking sounds, and it seems to be an idiomatic expression in Finnish language. In addition, both trigger words mean that the patient has difficulties maintaining the normal muscle tone of the upper respiratory track, which, in turn, reflects a markedly altered level of consciousness. The latter trigger word was associated with a later confirmed non-cardiac arrest event, whereas the former was the most frequently used single trigger word in the confirmed true cardiac arrest stratum.

As discussed above, the emergency dispatcher had missed two cases, in which ambulance personnel encountered cardiac arrest. Interestingly, ‘is wheezing’ was the only spontaneous trigger word in the first missed case. The second case included the following trigger words: ‘shallow breathing’, ‘I’m not sure if the patient is breathing’ and ‘glazed eyes’. These trigger words may reflect agonal breathing, which seems to be a pitfall of recognition of OHCA [21]. Indeed, subtle changes to the current algorithm may result in better sensitivity without a marked decrease in specificity. Riou et al. suggested that the emergency dispatcher should repeat the question regarding breathing pattern if the caller’s initial answer is imprecise or vague [16].

In the future, trigger word combinations could be identified in real time by automatic speech recognition, and machine-learning models could calculate a probability of cardiac arrest. Corti AI, used by emergency dispatchers in Denmark, is an example of an automatic speech recognition program [22]. A recently published study evaluated this machine-learning algorithm to emergency dispatchers and showed that Corti AI seems to outperform emergency dispatchers for recognising OHCA [23].

### Strengths and limitations

To the best of our knowledge, no previous study focusing on recognition of OHCA has explored spontaneous speech in emergency calls. Besides novelty, the strength of the study is the contribution of two native Finnish linguists, which increases the potential generalisability of the results beyond Finland.

This descriptive pilot study has several important limitations to consider. First, the study failed to detect any association between confirmed cardiac arrests and trigger words, and the confidence intervals for odds ratios were wide in the logistic regression model. However, this study was designed as a pilot study. A further study with a greater sample size is currently being conducted. Second, the study was underpowered to find trigger words associated with false negative cases (i.e. the dispatcher may have not suspected OHCA, even though a true cardiac arrest had occurred). This was a rare event in our material, as the dispatcher had missed only two later confirmed OHCAs. Third, the authors were not blinded to the outcome when transcribing the emergency calls or categorising the trigger words. Fourth, the exact time of trigger words and the time of OHCA suspicion in an emergency call were not considered in our analysis. However, this study was not designed to address trigger words associated with prompt or late recognition of OHCA. Finally, transportation codes were used to confirm cardiac arrest. Nevertheless, it is extremely rare that EMS personnel would have used the transportation codes of OHCA for non-cardiac arrest missions and vice versa.

## Conclusions

In conclusion, this pilot study introduces a novel method to categorise laypeople’s spontaneous trigger words in emergency calls in the context of dispatcher-suspected cardiac arrest. No trigger word was associated with confirmed cardiac arrests, but ‘is wheezing’ was the most frequent trigger word in the confirmed cardiac arrest stratum.

## Data Availability

The data that support the findings of this study (including the complete list of Finnish trigger words with their English translations) are available from the corresponding author upon request.

## Abbreviations

CI: Confidence interval
CPR: Cardiopulmonary resuscitation
ELS: EinsatzLeitSystem
EMS: Emergency medical services
ERC: European Resuscitation Council
ILCOR: International Liaison Committee on Resuscitation
OHCA: Out-of-hospital cardiac arrest
OR: Odds ratio

## References

[1] Gräsner JT, Lefering R, Koster RW, Masterson S, Böttiger BW, Herlitz J (2016). EuReCa ONE—27 nations, ONE Europe, ONE registry: a prospective one month analysis of out-of-hospital cardiac arrest outcomes in 27 countries in Europe. Resuscitation.

[2] Berdowski J, Berg RA, Tijssen JG, Koster RW (2010). Global incidences of out-of-hospital cardiac arrest and survival rates: systematic review of 67 prospective studies. Resuscitation.

[3] Kitamura T, Iwami T, Kawamura T, Nitta M, Nagao K, Nonogi H (2012). Nationwide improvements in survival from out-of-hospital cardiac arrest in Japan. Circulation.

[4] Hasselqvist-Ax I, Riva G, Herlitz J, Rosenqvist M, Hollenberg J, Nordberg P (2015). Early cardiopulmonary resuscitation in out-of-hospital cardiac arrest. N Engl J Med.

[5] Kragholm K, Wissenberg M, Mortensen RN, Hansen SM, Malta Hansen C, Thorsteinsson K (2017). Bystander efforts and 1-year outcomes in out-of-hospital cardiac arrest. N Engl J Med.

[6] Perkins GD, Handley AJ, Koster RW, Castrén M, Smyth MA, Olasveengen T (2015). European resuscitation council guidelines for resuscitation 2015. Section 2Adult basic life support and automated external defibrillation. Resuscitation.

[7] Berdowski J, Beekhuis F, Zwinderman AH, Tijssen JGP, Koster RW. Importance of the first link: description and recognition of an out-of-hospital cardiac arrest in an emergency call. 2009;119:2096–102. 10.1161/CIRCULATIONAHA.108.768325 19349324

[8] Viereck S, Møller TP, Ersbøll AK, Bækgaard JS, Claesson A, Hollenberg J (2017). Recognising out-of-hospital cardiac arrest during emergency calls increases bystander cardiopulmonary resuscitation and survival. Resuscitation..

[9] Olasveengen TM, de Caen AR, Mancini ME, Maconochie IK, Aickin R, Atkins DL (2017). 2017 international consensus on cardiopulmonary resuscitation and emergency cardiovascular care science with treatment recommendations summary. Resuscitation.

[10] Official statistics of Finland (OSF): Population structure. 2017. http://www.stat.fi/til/vaerak/index_en.html. Accessed 2 July 2019.

[11] Nurmi J, Pettilä V, Biber B, Kuisma M, Komulainen R, Castrén M (2006). Effect of protocol compliance to cardiac arrest identification by emergency medical dispatchers. Resuscitation.

[12] Pesonen J. Arvioinnin kehittäminen hätäkeskuspäivystäjien tutkinnossa. 2009. https://www.theseus.fi/bitstream/handle/10024/8102/Pesonen.Jouko.pdf?sequence=2. Accessed 2 July 2019.

[13] Mäkelä, K. Kvalitatiivisen aineiston analyysi ja tulkinta [Analysis and Interpretation of the Qualitative Data]. Helsinki: Gaudeamus; 1990.

[14] Krippendorf, K. Content analysis: an introduction to its methodology. 2nd ed. Thousand Oaks, California: Sage Publications; 2004.

[15] The ABCE approach. 2019.https://www.resus.org.uk/resuscitation-guidelines/abcde-approach. Accessed 20 Oct 2019.

[16] Riou M, Ball S, Williams TA, Whiteside A, Cameron P, Fatovich DM (2018). ‘She’s sort of breathing’: what linguistic factors determine call-taker recognition of agonal breathing in emergency calls for cardiac arrest?. Resuscitation.

[17] Riou M, Ball S, Whiteside A, Bray J, Perkins GD, Smith K (2018). ‘We’re going to do CPR’: a linguistic study of the words used to initiate dispatcher-assisted CPR and their association with caller agreement. Resuscitation.

[18] Viereck S, Møller TP, Rothman JP, Folke F, Lippert FK (2017). Recognition of out-of-hospital cardiac arrest during emergency calls - a systematic review of observational studies. Scand J Trauma Resusc Emerg Med.

[19] Hiltunen PVC, TO S, Jäntti TH, Kuisma MJ, Kurola JO (2015). Emergency dispatch process and patient outcome in bystander-witnessed out-of-hospital cardiac arrest with a shockable rhythm. Eur J Emerg Med.

[20] Kuisma M, Boyd J, Väyrynen T, Repo J, Nousila-Wiik M, Holmström P (2005). Emergency call processing and survival from out-of-hospital ventricular fibrillation. Resuscitation.

[21] Fukushima H, Imanishi M, Iwami T, Seki T, Kawai Y, Norimoto K (2014). Abnormal breathing of sudden cardiac arrest victims described by laypersons and its association with emergency medical service dispatcher-assisted cardiopulmonary resuscitation instruction. Emerg Med J.

[22] Artificial Intelligence that saves lives. 2019. https://corti.ai. Accessed 2 July 2019.

[23] Blomberg SN, Folke F, Ersbøll AK, Christensen HC, Torp-Pedersen C, Sayre MR (2019). Machine learning as a supportive tool to recognize cardiac arrest in emergency calls. Resuscitation.

